# A Two-decade Assessment of Changing Practice for Surgical Decompression and Fixation after Traumatic Spinal Cord Injury – Impact on Healthcare Utilization and Cost

**DOI:** 10.7759/cureus.6156

**Published:** 2019-11-14

**Authors:** Beatrice Ugiliweneza, James Guest, April Herrity, Miriam Nuno, Mayur Sharma, Jennifer Beswick, Nicholas Dietz, Ahmad Alhourani, Dengzhi Wang, Doniel Drazin, Maxwell Boakye

**Affiliations:** 1 Neurosurgery, University of Louisville School of Medicine, Louisville, USA; 2 Neurosurgery, The Miami Project to Cure Paralysis, University of Miami, Miami, USA; 3 Statistics, University of California, Davis, USA; 4 Medicine, Pacific Northwest University of Health Sciences, Yakima, USA

**Keywords:** traumatic spinal cord injury, surgery timing, outcomes, healthcare utilization, cost

## Abstract

Early surgery after traumatic spinal cord injury (TSCI) has been associated with a greater neurological recovery and reduced secondary complications. In this study, we aimed to evaluate the trend of early TSCI surgery (within 24 hours) over two decades and the effect on length of hospitalization, complications, and hospital charges. We extracted emergency admissions of adults diagnosed with TSCI from the National Inpatient Sample database (1998-2016). We analyzed the trend of early surgery and concurrent trends of complication rate, length of stay (LOS) and hospital charges. These outcomes were then compared between early and late surgery cohorts. There were 3942 (53%) TSCI patients who underwent early surgery, and 3446 (47%) were operated after 24 hours. The combined patient group characteristics consisted of median age 43 years (IQR: 29-59), 73% males, 72% white, 44% private payer, 18% Medicare, 17% Medicaid, 51% cervical, 30% thoracic, 75% from large hospitals, and 79% from teaching hospitals. The trend of early surgery, adjusted for annual case-mix, increased from 45% in 1998 to 64% in 2016. Each year was associated with 1.60% more patients undergoing early surgery than the previous year (*p*-value <0.05). During these years, the total LOS decreased, while hospital charges increased. Patients who underwent early surgery spent four fewer days in the hospital, accrued $28,705 lower in hospital charges and had 2.8% fewer complications than those with delay surgery. We found that the rate of early surgery has significantly increased from 1998 to 2016. However, as of 2016, one-third of patients still did not undergo spinal surgery within 24 hours. Late surgery is associated with higher complications, longer stays, and higher charges. The causes of delayed surgery are undoubtedly justified in some situations but require further delineation. Surgeons should consider performing surgery within 24 hours on patients with TSCI whenever feasible.

## Introduction

Traumatic spinal cord injury (TSCI) is a life-changing event resulting in serious functional, psychological, and socioeconomic consequences. In 2018, it was estimated that spinal cord injury (SCI) had an incidence estimated at 17,700 new cases annually with an overall prevalence of 288,000 persons living with SCI [1]. Chronic secondary medical complications are common sequelae causing increased morbidity, mortality, and decreased quality of life [2]. Over the decades, the treatment approach has changed markedly from the use of long-term bed rest to early surgical decompression and stabilization, mobilization, and early transfer to rehabilitation [3]. Early surgical intervention for TSCI (<24 hours) is gradually being more broadly translated into actual surgical practice [4]. While the timing of TSCI surgery has been debated in the literature since the early 1990s, the benefits of early surgery have been documented in several studies [5]. Early decompression has been associated with improved neurological and functional outcomes and decreased complications [6-8]. Despite the evidence, rates of patients undergoing early surgery have traditionally been low, and it is unclear if the rate of early surgery is increasing [9]. Concerns remain for safety of procedure for cervical central cord and efficacy for thoracic injuries. This study evaluated trends of early TSCI surgery over the past two decades to determine whether earlier surgery correlated with improved outcomes, healthcare utilization, and cost reduction. We hypothesized an increasing trend towards early surgery, particularly after 2012 when the Surgical Timing in Acute Spinal Cord Injury Study (STASCIS) was published [10]. We also hypothesized that as the application of early surgery increased, complications, healthcare utilization, and costs would decrease. As secondary analyses, we compare outcomes between early and late surgery.

## Materials and methods

Data sources

We used the years 1998-2016 from the National Inpatient Sample (NIS), an administrative discharge-level database sponsored by the Agency for Healthcare Research and Quality. To date, NIS is the largest all-payer inpatient care database with annually released data available from 1988 [11]. 

Study Population

This study includes adults aged 18 and older who were hospitalized and diagnosed with TSCI. Cases were extracted according to the International Classification of Diseases (ICD-9/10) codes for TSCI (cervical, thoracic, and lumbar) and concurrent surgical decompression codes (Supplemental Table 3). We defined early versus late spinal decompression surgery as decompression before or after 24 hours from admission. The 24-hour threshold was selected based on the STASCIS prospective clinical study [10]. Cases with missing time-to-surgery were excluded. 

Patient, injury, and hospital characteristics

Patient characteristics included age, gender, injury year, payer, and comorbidities, all noted at baseline. Five categories of payers were included in the analysis (Private, Medicaid, Medicare, Self-pay, and Other). Comorbidities were measured with the Elixhauser comorbidity score and computed using an adaptation to ICD-9/10 codes developed by Quan et al. [12-13]. Those cases lacking descriptors were excluded.

Injury characteristics were level of injury (cervical, thoracic and lumbar) and injury severity. Injury severity was captured through ICD-based injury severity scores (ICISS). ICISS was created as a data-driven survival score proxy to the injury severity score (ISS) [14]. ICISS varies from 0-1 with larger values representing a higher probability of survival.

Hospital characteristics included hospital bed numbers (small, medium, large), hospital region (Northeast, MidWest/North Central, South, and West), hospital location, and/or teaching status (rural, urban non-teaching, urban teaching). 

Outcome measures

The main outcomes of interest were in-hospital complications, total length of stay (LOS), LOS after surgery, and total charges. LOS after surgery was calculated as the difference between total LOS and the number of days from admission to surgery. Complications considered included renal, cardiac, general nervous system, general neurological, deep vein thrombosis or pulmonary embolism (DVT/PE), other pulmonary, infection, wound infection, pneumonia, acute kidney injury, pressure ulcers, and sepsis (Supplemental Table 4). All charges were inflated to 2016 U.S. dollars using the medical component of the consumer price index [15]. 

Statistical analysis

Analysis of Trend

The TSCI population has changed over the years (Supplemental Table 3). The generalized linear model characteristic-adjusted early surgery rates and outcomes were used in the analysis of trends instead of the raw values. The early surgery rates were analyzed using JoinPoint Regression software (Version 4.6.0.0 - April 2018; Statistical Methodology and Applications Branch, Surveillance Research Program, National Cancer Institute). Then, outcomes were plotted over time, compared with the early surgery rates to qualitatively evaluate the association of trends during the study period. The characteristic-adjusted values were also used in a secondary analysis qualitatively evaluating the association between days to surgery and outcomes. 

Comparison of Outcomes between Early and Late Surgery

Herein, the comparison of outcomes between early and late surgery has been summarized over two decades. To account for the characteristic differences between early and late surgery groups within each year (Supplemental Table 5), we used inverse probability of treatment weight (IPTW) method to balance the covariates. We used the annual IPTW weighted estimates and standard errors (SE) were pooled to obtain overall comparative estimates of the two groups over the two decades. 

Statistical Test Details and Software

Statistical analyses were performed in SAS (SAS Institute, Cary, NC). All tests were two-sided with *p* = 0.05. 

## Results

Description of patients in sample

In total, 7,388 adult TSCI patients underwent decompressive surgery for SCI between1998-2016. Among these, 3942 (53%) underwent early surgery while 3446 (47%) had surgery after 24 hours. Distributions of race, comorbidities, and patient residence zip code median income-quartiles were statistically comparable. Most patients sustained cervical injury (51%), followed by thoracic (30%) and then lumbar (19%). There were a greater number of cervical injuries in the early surgery group (54% vs. 49%, *p *< 0.05). The late surgery group included younger patients (median age 43 vs. 46, *p *< 0.05), more females (29% vs. 26%, *p *< 0.05) and more patients covered by Medicare (21% vs. 15%, *p* < 0.0001) (Table 1). IPTW was used to balance the covariates between groups within each year. 

**Table 1:** Patient, injury, and hospital characteristics for traumatic spinal cord injury patients injured between 1998 and 2016 ICISS, ICD-based injury severity score; SD, standard deviation; IQR, interquartile range

Variables		Late Surgery	Early Surgery		Combined
		(n = 3446)	(n = 3942)	p-value	(n = 7388)
	Mean (SD)	45 (19)	47 (20)		45 (19)
Age	Median (IQR)	43 (29, 59)	46 (30, 61)	<0.0001>	43 (29-59)
	Range, min-max	18 to 111	18 to 108		18 to 111
Sex	Males, n (%)	2461 (71%)	2902 (74%)	0.03	5363 (73%)
	Females, n (%)	985 (29%)	1040 (26%)		2025 (27%)
	White, n (%)	5346 (72%)	2472 (72%)		5346 (72%)
Race	Black, n (%)	924 (13%)	466 (13%)	0.04	924 (13%)
	Other, n (%)	1118 (15%)	508 (15%)		1118 (15%)
	1^st^ quartile, n (%)	1003 (29%)	1093 (28%)		2096 (28%)
Income	2^nd^ quartile, n (%)	952 (28%)	1057 (27%)	0.20	2009 (27%)
quartile	3^rd^ quartile, n (%)	795 (23%)	925 (23%)		1720 (23%)
	4^th^ quartile, n (%)	696 (20%)	867 (22%)		1563 (21%)
	Medicare, n (%)	711 (21%)	588 (15%)		1299 (18%)
Payer	Medicaid, n (%)	565 (16%)	716 (18%)		1281 (17%)
	Private, n (%)	1480 (43%)	1804 (46%)	<0.0001>	3284 (44%)
	Self-pay, n (%)	343 (10%)	387 (10%)		730 (10%)
	Other, n (%)	347 (10%)	447 (11%)		794 (11%)
	0, n (%)	870 (25%)	1080 (27%)		1950 (26%)
Elixhauser score	1, n (%)	935 (27%)	1036 (26%)	0.21	1971 (27%)
	2, n (%)	740 (21%)	835 (21%)		1575 (21%)
	3+, n (%)	901 (26%)	991 (25%)		1892 (26%)
	Mean (SD)	0.8 (0.2)	0.8 (0.2)		0.8 (0.2)
ICISS	Median (IQR)	0.8 (0.7, 0.9)	0.8 (0.7, 0.9)	0.0006	0.8 (0.7, 0.9)
	Range, min-max	0 to 1	0 to 1		0 to 1
Level of Injury	Cervical, n (%)	1687 (49%)	2111 (54%)		3798 (51%)
	Thoracic, n (%)	1106 (32%)	1080 (27%)	<0.0001>	2186 (30%)
	Lumbar, n (%)	653 (19%)	751 (19%)		1404 (19%)
Hospital Bed Size	Small, n (%)	95 (3%)	139 (4%)		234 (3%)
	Medium, n (%)	819 (24%)	758 (19%)	<0.0001>	1577 (21%)
	Large, n (%)	2532 (73%)	3045 (77%)		5577 (75%)
	Northeast, n (%)	754 (22%)	906 (23%)		1660 (22%)
Hospital Region	Midwest/Northcentral, n (%)	492 (14%)	639 (16%)	<0.0001>	1131 (15%)
	South, n (%)	1884 (55%)	1905 (48%)		3789 (51%)
	West, n (%)	316 (9%)	492 (12%)		808 (11%)
Hospital Urban/Teaching	Rural, n (%)	90 (3%)	110 (3%)		200 (3%)
	Urban Non-teaching, n (%)	667 (19%)	656 (17%)	0.01	1323 (18%)
	Urban Teaching, n (%)	2689 (78%)	3176 (81%)		5865 (79%)

Trend of early surgery

**Figure 1:**
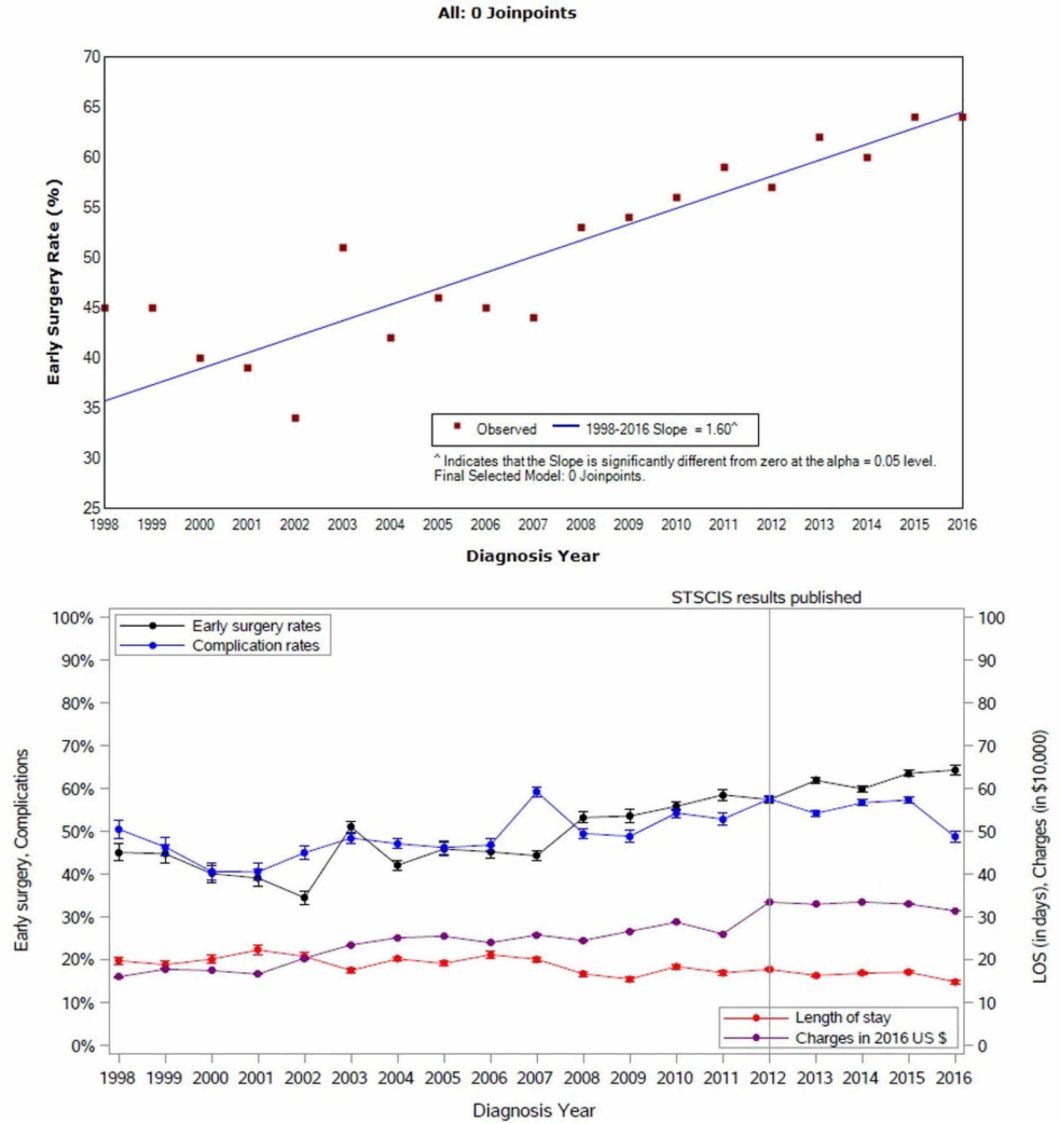
Early surgery rate and year and early surgery complications from 1998 to 2016

The proportion of early surgery increased from approximately 45% in 1998 to approximately 64% in 2016 (Figure 1). This trend has been consistently increasing: each year, more patients (1.60%) underwent early surgery than the previous year. Interestingly, the STASCIS study that indicated greater effectiveness of early surgery did not appear to alter this trend. Results from JoinPoint regression analysis show no break in the trend 1998-2016. 

Trends in outcomes and correlation with trend in early surgery

Over the years analyzed, the percentage of patients experiencing at least one complication while hospitalized slowly decreased starting at 51% in 1998 and ending with 49% in 2016. The LOS decreased from an average of 22 days (SE: 2.92) to 15 days (SE: 0.91). Despite this reduction, hospital charges increased from an average of $161,510 (SE: $20,459) in 1998 to $317,910 (SE: $18,037) in 2016. 

Comparison of outcomes, early versus late surgery

Late surgery was associated with more complications, longer total hospital stays and higher charges (Table 2). These differences were consistently observed throughout most years. The 2 groups had similar hospital stays after surgery. In the pooled analysis of IPTW cohorts, late surgery SCI patients had an average longer stay (20 vs. 16 days, *p *< 0.0001). This difference was attributed to time prior to surgery since LOS after surgery was statistically comparable (about 15 days, *p *= 0.75). The hospital charges were higher for late surgery patients ($287,576 vs. $258,871, *p* = 0.0003) who also had more complications: nervous system (3% vs. 1%, *p *< 0.0001), infection (13.6% vs. 11.8%, *p =* 0.03), pneumonia (19.1% vs. 16.5%, *p =* 0.01), pressure ulcers (7.18% vs. 5.29%, *p* = 0.0002), sepsis (4.12% vs. 3.21%, *p* = 0.03). Other complications (renal, cardiac, neurological, DVT/PE, pulmonary, wound, acute kidney failure) were similar. Late surgery patients were more likely to experience at least one complication evaluated (53.2% vs. 50.4%, *p* = 0.02).

**Table 2:** Outcomes (IPTW - weighted values) SE, standard error; IPTW, inverse probability of treatment weight

	Late Surgery																																																																																																																							Early Surgery																																																										p-value																						Combined
	(n = 3446)																																																																																																																							(n = 3942)																																																																																
In-hospital length of stay and charges																																																																																																																																																																																																								
Length of stay (days), mean (SE)	20.5 (0.63)																																																																																																																							15.8 (0.67)																																																										<0.0001>																						17.9 (0.31)
Days after surgery, mean (SE)	15.1 (0.58)																																																																																																																							15.3 (0.66)																																																										0.75																						15.2 (0.30)
Charges (2016 US $), median (SE)	287576 (7600)																																																																																																																							258871 (8375)																																																										0.0003																						272220 (3362)
Complications*																																																																																																																																																																																																								
Renal, % (SE)	1.11% (0.27%)																																																																																																																							1.00% (0.24%)																																																										0.67																						1.05% (0.12%)
Cardiac, % (SE)	1.44% (0.30%)																																																																																																																							0.69% (0.57%)																																																										0.11																						1.32% (0.14%)
Neurological, % (SE)	0.32% (0.16%)																																																																																																																							0.32% (0.12%)																																																										1.00																						0.32% (0.07%)
Nervous System, % (SE)	3.15% (0.42%)																																																																																																																							1.03% (0.38%)																																																										.0001																						3.09% (0.20%)
Deep venous thrombosis/pulmonary embolus, % (SE)	6.87% (0.62%)																																																																																																																							6.02% (0.56%)																																																										0.15																						6.42% (0.29%)
Pulmonary, % (SE)	35.0% (1.19%)																																																																																																																							36.2% (1.14%)																																																										0.30																						35.6% (0.57%)
Infection, % (SE)	13.6% (0.86%)																																																																																																																							11.8% (0.80%)																																																										0.03																						12.7% (0.41%)
Wound, % (SE)	1.15% (0.27%)																																																																																																																							0.66% (0.48%)																																																										0.22																						0.89% (0.12%)
Pneumonia, % (SE)	19.1% (1.00%)																																																																																																																							16.5% (0.95%)																																																										0.01																						17.7% (0.47%)
Acute kidney failure, % (SE)	5.11% (0.52%)																																																																																																																							4.86% (0.27%)																																																										0.54																						4.98% (0.24%)
Pressure ulcers, % (SE)	7.18% (0.65%)																																																																																																																							5.29% (0.32%)																																																										0.0002																						6.17% (0.30%)
Sepsis, % (SE)	4.12% (0.45%)																																																																																																																							3.21% (0.38%)																																																										0.03																						3.63% (0.20%)
At least one of the above, % (SE)	53.2% (1.26%)																																																																																																																							50.4% (1.22%)																																																										0.02																						51.7% (0.61%)

Effect of days to surgery on outcomes

For the first three days, the complication rate curve remained constant at 48%, but by day 5, complication rates rose to mid-50%. As the surgery was further delayed, complication rates increased, slowly reaching mid-90% after about four weeks. LOS and hospital charges were marginally impacted by the surgical delay of up to 1.5 weeks. Starting at day 10, the later surgery was performed, the higher the LOS and associated hospital charges. 

## Discussion

The trend towards early surgery consistently increased from 1998 to 2016 at an annual rate of 1.60%. Of note, this trend was statistically the same before and after 2012, when results from the STASCIS study were published. LOS decreased and hospital charges increased. Compared to late surgery patients, early surgery patients had consistently better outcomes in most years, averaging five less hospital days, $28,705 less in hospital charges and 2.8% reduced risk of complications.

Trends of surgical timing

Traditionally, early decompression has been questioned for benefit of neurological recovery and its association with complication rates, given that early surgery was associated with worse neurological outcomes [16]. During the study interval, imaging, anesthesia, spinal instrumentation, and intensive care practices have seen great improvements that have reduced surgical risks. 

Earlier analyses documented lowest early surgery rates, whereas studies from more recent years had the highest early surgery rates [4,7,17-19]. Our study is the first, to our knowledge, to evaluate the year-to-year trend of early spinal decompression over the past two decades. In the overall combined population, we found early decompression of 53%. This is consistent with studies spanning the years analyzed: early surgery rates ranged from 21% to 58%. We found that from 1998 to 2016, early surgery increased by 1.60% annually. Our hypothesis that the trend of early surgery after the publication of the STASCIS study would be higher than the prior trend was not supported by the data. STASCIS was a multicenter prospective study that provided strong support that early surgery is beneficial. From our analysis, the early surgery rates appeared to have plateaued in 2013 as compared to previous years at approximately 63%. An increasing body of evidence has supported that compression of the spinal cord should be treated as an emergency. However, in practice, this may be difficult to accomplish given low staffing outside of working hours, the need to disrupt preplanned surgical schedules, access to MRI, and the variability of TSCI. This observation that STASCIS did not dramatically shift the trend for early surgery likely derives from multiple factors. Future studies should evaluate if this trend begins to increase again after 2016. In 2016, one-third of TSCI surgical patients did not undergo surgery within 24 hours. In a systematic review of the timing of TSCI decompression, Wilson et al. showed that although the benefits of early surgery in cervical TSCI have been supported, for the non-cervical regions, the evidence is still debatable [20]. Current guidelines recommend that for TSCI cases presenting with central cord syndrome, decompression within 24 hours should be considered as an option [21]. For all other TSCI cases, early surgery (without a specific time threshold) is suggested as an option to reduce LOS and complications [21-22]. Given the apparent benefits of early surgery, future studies should explore the factors associated with delayed surgery. Potential reasons include patient factors such as polytrauma and comorbidities as well as institutional factors such as operating room availability and hospital size.

Complications

Complications during the acute SCI period have been established as disease-modifying events that reduce recovery [23]. Bourassa-Moreau et al. compared complication rates between early and late surgery patients [24]. They found the early surgery group had fewer cases of pneumonia (20% vs. 36%), pressure ulcers (16% vs. 25%), and global complications (42% vs. 63%). Liu et al. conducted a meta-analysis finding that early surgery was associated with a 40% reduced risk of experiencing complications (odds ratio: 0.61, 95% CI: 0.40-0.91) [22]. Data from our study are consistent with these earlier reports. We found that, compared to late surgery, early surgery patients had lower complications of pneumonia (19% vs 17%) and pressure ulcers (7% vs. 5%). This may be due to different population demographics, their study included only complete TSCI while ours included all TSCI. Complete injuries are more severe and therefore more likely to be associated with complications post-injury [25]. Fifty percent of early surgery patients experienced at least one complication versus 53% for late surgery patients. Among other complications we evaluated, early surgery was associated with less nervous system complications, infections, wound problems, and sepsis. 

Hospital length of stay and charges

We found early surgery to be associated with five-day shorter stays and $28,705 lower charges. This is consistent with prior studies evaluating early surgery outcomes. Mac-Thiong et al. found that early surgery SCI patients have nine-day shorter LOS [6]. From meta-analysis, Liu et al. found that difference to be five days [22]. Regarding cost, Furlan et al. conducted a cost-effectiveness analysis showing that early decompression is more cost-effective compared to delayed decompression in both complete SCI ($524,484 versus $544,852 per Quality-Adjusted Life Year (QALY) gained) and incomplete SCI ($82,008 vs $91,233 per QALY gained) [26]. Two Canadian studies found that patients decompressed within 24 hours had about 4,000 Canadian dollars lower charges than those with later spinal surgery in all TSCI and 7,000 Canadian dollars less in complete TSCI [24]. Of note, the relative days counted from the day of surgery to discharge did not differ between the early and late surgery groups. Hence, the difference observed in total LOS was likely due to the delay of surgery. Reducing this time to surgery could potentially save hospitalization days and the associated expenditures. We found that the average LOS for all patients undergoing TSCI surgery has decreased over the years examined. Never-the-less the total charges increased consistently. This increase may be attributable to increasing healthcare costs in society and not necessarily the timing of surgery [21,27]. The improvement in surgical care with the advances in imaging and spinal fixation hardware might have contributed to the increase in the cost while enabling reduced LOS. 

Association of delay to surgery with complications and healthcare utilization

Our data suggests stable complication rates if surgery is performed within the first three days. Complications that were significantly different with surgery delayed beyond three days were related to the nervous system, infection, pneumonia, and sepsis, most of which take a few days to develop. Based on the risk of complications, our data support that if spinal surgery is not performed in 24 hours, it should occur within 72 hours. 

Strengths and limitations of this study

NIS is a large database and representative sample of U.S. hospitalizations documenting real-world practices in U.S. healthcare and an excellent resource for health services research. However, there are some limitations: NIS is limited to hospitalization; it is not possible to inquire about the resources used or clinical outcomes prior to the injury hospitalization or to follow up patients after discharge. Although complications were identified, we could not determine their severity. Given that NIS is discharge-level data, it consists of claims-based data relying on ICD-9 and ICD-10 codes to define cohorts. There is the possibility of coding error, although minimal [28]. Completeness of injury was not factored into the analysis because ICD-9 coding does not distinguish complete and incomplete in the lumbar region. Although we used ITPW to balance observed confounders, there might be some residual selection bias given the observational study design. Despite these limitations, this study provides a long-term assessment of trends in spinal surgery timing for TSCI. Given the inherent difficulty to conduct randomized studies in this population, these results add additional evidence that delay of surgery should be carefully weighed against the increased risk of complications and reduced neurological recovery.

## Conclusions

Early surgery to decompress and stabilize the spine has emerged as an accepted standard that may improve neurological outcome and reduce acute care duration in many instances. This study shows while the rate of early surgery has increased, as of 2016, about 30% of patients did not undergo surgery within 24 hours. Early surgery was associated with fewer complications, shorter hospital stays, and lower charges. Beyond three days after admission, the more days that pass before surgery, the higher the risk of complications. Future studies should identify the causes of a delay in surgery to determine if some can be mitigated. Given the established benefits of earlier surgery for TSCI, surgeons should prioritize surgery within 24 hours in the absence of compelling reasons to delay. 

## Appendices

**Table 3:** International Classification of Diseases 9/10 codes used for case extraction and complications

		International Classification of Diseases-9	International Classification of Diseases-10
Diagnosis	Cervical	806.0, 806.1	S14.0--A or S14.1—A + S12.---A
Of closed	Thoracic	806.2, 806.3	S24.0--A or S24.1—A + S22.---A
SCI	Lumbar	806.4, 806.5	S34.0--A or S34.1—A + S32.---A
Surgery = Decompression		03.01, 03.09, 03.4, 03.53	00CW, 00CX, 00CY, 0PN3, 0PN4, 0SN0, 0SN2, 0SN3, 0SN4, 0PB3, 0PB4, 0SB0, 0SB2, 0SB3, 0SB4, 00BW, 00BX, 00BY, 005W, 005X, 00WY, 0PS3, 0PS4, 0QS0, 0RBA, 0RBB, 0SB0, 0SB1, 0SB2, 0SB3, 0SB4, 0R5A, 0R5B, 0S50, 0S51, 0S52, 0S53, 0S54, 0QB0, 0Q50, 0SB6, 0SB4, 0RB1, 0SB3, 0SB0
	Renal	583, 997.5	N17, N9989
	Cardiac	410, 997.1	I21, I977, I978
	Nervous system	997.0	G97
	Neurological	430-436, 438.2, 438.3, 438.4, 438.5	I60, I61, I62, I63, I64, I65, I66, I67, I68, I69
	Deep venous thrombosis/Pulmonary Embolus	415, 451, 452, 453	I26, I80, I81, I82
Complications	Pulmonary	518.4, 518.5, 518.8, 997.3	J810, J80, J951, J952, J953, J958, J96
	Infection	595.0, 595.9, 599.0	N3000, N3001, N3090, N3091, N390
	Wound	998.32, 998.51, 998.6, 998.81, 998.83	T8131, T814, T818
	Pneumonia	481, 482, 486	J13, J14, J15, J16, J17, J18
	Kidney failure	584,	N17
	Pressure ulcers	707,	L89
	Sepsis	995.91, 995.92	A4181, A419, R652

**Table 4:** Patient, injury, and hospital characteristics from 1998 to 2016 *, *p*-value < 0.05; Sx^a^,^ ^Surgery; N, Northeast; MW/NC, Midwest/Northcentral; S, South; W, West; SD, standard deviation

Variables		Selected Years Between 1998 and 2016						All Years Between 1998 and 2016			
		1998	2000	2004	2008	2012	2016	Combined	Late Sx^a^	Early Sx^a^	
		(n = 202)	(n = 212)	(n = 395)	(n = 374)	(n = 716)	(n = 370)	(n = 7388)	(n = 3446)	(n = 3942)	
	Mean (SD)	40 (18)	40 (17)	41 (17)	46 (20)	47 (19)	46 (19)	45 (19)	45 (19)	47 (20)	
Age	Median (interquartile range)	35 (25, 51)	36 (28, 50)	37 (27, 52)	44 (28, 59)	48 (30, 60)	45 (28, 61)	43 (29-59)	43 (29, 59)	* 46 (30, 61)	
	Range, min-max	18 to 88	18 to 84	18 to 90	18 to 91	18 to 90	18 to 90	18-111	18 to 111	18 to 108	
Gender	Males, n (%)	152 (75%)	149 (70%)	281 (71%)	249 (67%)	520 (73%)	258 (70%)	5363 (73%)	2461 (71%)	2902 (74%)	*
	Females, n (%)	50 (25%)	63 (30%)	114 (29%)	125 (33%)	196 (27%)	112 (30%)	2025 (27%)	985 (29%)	1040 (26%)	
	White, n (%)	158 (78%)	167 (79%)	283 (72%)	288 (77%)	531 (74%)	258 (70%)	5346 (72%)	5346 (72%)	2472 (72%)	
Race	Black, n (%)	32 (16%)	24 (11%)	51 (13%)	39 (10%)	82 (11%)	33 (9%)	924 (13%)	924 (13%)	466 (13%)	
	Other, n (%)	12 (6%)	21 (10%)	61 (15%)	47 (13%)	103 (14%)	79 (21%)	1118 (15%)	1118 (15%)	508 (15%)	
	1^st ^quartile, n (%)	18 (9%)	13 (6%)	126 (32%)	119 (32%)	225 (31%)	118 (32%)	2096 (28%)	1003 (29%)	1093 (28%)	
Income Quartile	2^nd ^quartile, n (%)	76 (38%)	82 (39%)	121 (31%)	107 (29%)	181 (25%)	80 (22%)	2009 (27%)	952 (28%)	1057 (27%)	
	3^rd ^quartile, n (%)	51 (25%)	58 (27%)	84 (21%)	73 (20%)	164 (23%)	87 (24%)	1720 (23%)	795 (23%)	925 (23%)	
	4^th ^quartile, n (%)	57 (28%)	59 (28%)	64 (16%)	75 (20%)	146 (20%)	85 (23%)	1563 (21%)	696 (20%)	867 (22%)	
	Medicare, n (%)	19 (9%)	17 (8%)	35 (9%)	60 (16%)	139 (19%)	89 (24%)	1299 (18%)	711 (21%)	588 (15%)	
Payer	Medicaid, n (%)	34 (17%)	32 (15%)	68 (17%)	72 (19%)	124 (17%)	75 (20%)	1281 (17%)	565 (16%)	716 (18%)	*
	Private, n (%)	111 (55%)	119 (56%)	207 (52%)	171 (46%)	318 (44%)	137 (37%)	3284 (44%)	1480 (43%)	1804 (46%)	
	Self-Pay, n (%)	12 (6%)	20 (9%)	45 (11%)	35 (9%)	66 (9%)	32 (9%)	730 (10%)	343 (10%)	387 (10%)	
	Other, n (%)	26 (13%)	24 (11%)	40 (10%)	36 (10%)	69 (10%)	37 (10%)	794 (11%)	347 (10%)	447 (11%)	
	0, n (%)	88 (44%)	81 (38%)	149 (38%)	104 (28%)	126 (18%)	79 (21%)	1950 (26%)	870 (25%)	1080 (27%)	
Elixhauser Score	1, n (%)	66 (33%)	63 (30%)	107 (27%)	108 (29%)	191 (27%)	102 (28%)	1971 (27%)	935 (27%)	1036 (26%)	
	2, n (%)	25 (12%)	48 (23%)	76 (19%)	69 (18%)	162 (23%)	84 (23%)	1575 (21%)	740 (21%)	835 (21%)	
	3+, n (%)	23 (11%)	20 (9%)	63 (16%)	93 (25%)	237 (33%)	105 (28%)	1892 (26%)	901 (26%)	991 (25%)	
	Mean (SD)	0.8 (0.2)	0.8 (0.2)	0.8 (0.2)	0.8 (0.2)	0.8 (0.2)	0.6 (0)	0.8 (0.2)	0.8 (0.2)	0.8 (0.2)	*
ICISS	Median (interquartile range)	0.8 (0.7, 0.9)	0.8 (0.7, 0.9)	0.8 (0.7, 0.9)	0.8 (0.7, 0.9)	0.8 (0.7, 0.9)	0.6 (0.6, 0.6)	0.8 (0.7, 0.9)	0.8 (0.7, 0.9)	0.8 (0.7, 0.9)	
	Range, min-max	0.1 to 1	0.4 to 1	0.2 to 1	0.1 to 1	0 to 1	0.6 to 0.6	0 to 1	0 to 1	0 to 1	
Level of Injury	Cervical, n (%)	76 (38%)	95 (45%)	187 (47%)	178 (48%)	395 (55%)	148 (40%)	3798 (51%)	1687 (49%)	2111 (54%)	*
	Thoracic, n (%)	77 (38%)	71 (33%)	123 (31%)	109 (29%)	210 (29%)	134 (36%)	2186 (30%)	1106 (32%)	1080 (27%)	
	Lumbar, n (%)	49 (24%)	46 (22%)	85 (22%)	87 (23%)	111 (16%)	88 (24%)	1404 (19%)	653 (19%)	751 (19%)	
Hospital Bed Size	Small, n (%)	2 (1%)	0 (0%)	2 (1%)	1 (0%)	26 (4%)	19 (5%)	234 (3%)	95 (3%)	* 139 (4%)	
	Medium, n (%)	58 (29%)	49 (23%)	76 (19%)	83 (22%)	120 (17%)	71 (19%)	1577 (21%)	819 (24%)	758 (19%)	
	Large, n (%)	142 (70%)	163 (77%)	317 (80%)	290 (78%)	570 (80%)	280 (76%)	5577 (75%)	2532 (73%)	3045 (77%)	
	N^a^, n (%)	50 (25%)	46 (22%)	98 (25%)	119 (32%)	113 (16%)	61 (16%)	1660 (22%)	754 (22%)	906 (23%)	
Hospital	MW/NC^a^, n (%)	39 (19%)	45 (21%)	35 (9%)	39 (10%)	132 (18%)	62 (17%)	1131 (15%)	492 (14%)	639 (16%)	*
Region	S^a^, n (%)	99 (49%)	112 (53%)	255 (65%)	198 (53%)	323 (45%)	160 (43%)	3789 (51%)	1884 (55%)	1905 (48%)	
	W^a^, n (%)	14 (7%)	9 (4%)	7 (2%)	18 (5%)	148 (21%)	87 (24%)	808 (11%)	316 (9%)	492 (12%)	
Hospital Urban/Teaching (T)	Rural, n (%)	21 (10%)	9 (4%)	13 (3%)	3 (1%)	6 (1%)	7 (2%)	200 (3%)	90 (3%)	110 (3%)	*
	Urban non-T, n (%)	22 (11%)	83 (39%)	58 (15%)	77 (21%)	122 (17%)	43 (12%)	1323 (18%)	667 (19%)	656 (17%)	
	Urban T, n (%)	159 (79%)	120 (57%)	324 (82%)	294 (79%)	588 (82%)	320 (86%)	5865 (79%)	2689 (78%)	3176 (81%)	

**Table 5:** Patient, injury, and hospital characteristics compared between early and late surgery from 1998 to 2016 *, *p*-value < 0.05; Sx^a^,^ ^surgery; N, northeast; MW/NC, midwest/northcentral; S, south; W, west; SD, standard deviation; ICISS, ICD-based injury severity scores

Variables		National Inpatient Sample 1998 data						National Inpatient Sample 2016 data					
		Unweighted data			IPTW^a^ weighted data			Unweighted data			IPTW^a^ weighted data		
		Late Sx^a^	Early Sx^a^		Late Sx^a^	Early Sx^a^		Late Sx^a^	Early Sx^a^		Late Sx^a^	Early Sx^a^	
		(n = 111)	(n = 91)		(n = 112)	(n = 90)		(n = 132)	(n = 238)		(n = 132)	(n = 238)	
	Mean (SD)	40 (18)	40 (19)		39 (17)	39 (18)		50 (20)	44 (19)		46 (20)	46 (19)
Age	Median (Interquartile Range)	36 (25, 51)	34 (26, 49)		35 (26, 48)	33 (26, 50)		51 (30, 66)	41 (27, 58)	*	45 (28, 62)	45 (28, 61)
	Range, min-max	18 to 85	18 to 88		18 to 85	18 to 88		18 to 90	18 to 90		18 to 90	18 to 90
Gender	Males, n (%)	83 (75%)	69 (76%)		88 (78%)	69 (77%)		89 (67%)	169 (71%)		93 (71%)	166 (70%)
	Females, n (%)	28 (25%)	22 (24%)		24 (22%)	21 (23%)		43 (33%)	69 (29%)		38 (29%)	71 (30%)
	White, n (%)	83 (75%)	75 (82%)		87 (78%)	68 (76%)		97 (73%)	161 (68%)		94 (71%)	165 (70%)
Race	Black, n (%)	21 (19%)	11 (12%)		17 (15%)	13 (14%)		9 (7%)	24 (10%)		11 (8%)	23 (10%)
	Other, n (%)	7 (6%)	5 (5%)		7 (6%)	9 (10%)		26 (20%)	53 (22%)		27 (20%)	50 (21%)
	1^st^quartile, n (%)	12 (11%)	6 (7%)		10 (9%)	9 (10%)		46 (35%)	72 (30%)		39 (30%)	76 (32%)
Income Quartile	2^nd^quartile, n (%)	44 (40%)	32 (35%)		44 (40%)	33 (37%)		26 (20%)	54 (23%)		30 (23%)	51 (21%)
	3^rd^quartile, n (%)	26 (23%)	25 (27%)		27 (24%)	24 (27%)		33 (25%)	54 (23%)		33 (25%)	56 (23%)
	4^th^quartile, n (%)	29 (26%)	28 (31%)		30 (27%)	24 (26%)		27 (20%)	58 (24%)		29 (22%)	55 (23%)
	Medicare, n (%)	14 (13%)	5 (5%)		11 (10%)	7 (8%)		40 (30%)	49 (21%)		31 (24%)	56 (23%)
Payer	Medicaid, n (%)	22 (20%)	12 (13%)		19 (17%)	22 (24%)		24 (18%)	51 (21%)		27 (20%)	48 (20%)
	Private, n (%)	56 (50%)	55 (60%)		60 (54%)	44 (49%)		49 (37%)	88 (37%)		50 (38%)	90 (38%)
	Self-pay, n (%)	7 (6%)	5 (5%)		7 (6%)	5 (6%)		6 (5%)	26 (11%)		9 (7%)	20 (9%)
	Other, n (%)	12 (11%)	14 (15%)		15 (14%)	11 (13%)		13 (10%)	24 (10%)		15 (11%)	24 (10%)
	0, n (%)	51 (46%)	37 (41%)		42 (38%)	33 (37%)		26 (20%)	53 (22%)		27 (20%)	50 (21%)
Elixhauser Score	1, n (%)	35 (32%)	31 (34%)		44 (39%)	34 (38%)		36 (27%)	66 (28%)		37 (28%)	66 (28%)
	2, n (%)	12 (11%)	13 (14%)		13 (12%)	11 (12%)		25 (19%)	59 (25%)		29 (22%)	53 (22%)
	3+, n (%)	13 (12%)	10 (11%)		13 (12%)	11 (13%)		45 (34%)	60 (25%)		38 (29%)	69 (29%)
	Mean (SD)	0.8 (0.2)	0.8 (0.2)		0.8 (0.2)	0.8 (0.2)		0.7 (0.2)	0.7 (0.2)		0.7 (0.2)	0.7 (0.2)
ICISS	Median (Interquartile Range)	0.8 (0.7, 0.9)	0.9 (0.8,0.9)		0.8 (0.7, 0.9)	0.9 (0.7, 0.9)		0.8 (0.6, 0.9)	0.7 (0.6, 0.9)		0.8 (0.6, 0.9)	0.7 (0.7, 0.9)
	Range, min-max	0.1 to 1	0.1 to 1		0.1 to 1	0.1 to 1		0.2 to 1	0.2 to 1		0.2 to 1	0.2 to 1
Level of Injury	Cervical, n (%)	35 (32%)	41 (45%)		47 (42%)	39 (43%)		46 (35%)	102 (43%)		54 (41%)	94 (40%)
	Thoracic, n (%)	51 (46%)	26 (29%)	*	39 (35%)	30 (33%)		53 (40%)	81 (34%)		46 (35%)	88 (37%)
	Lumbar, n (%)	25 (23%)	24 (26%)		26 (23%)	21 (24%)		33 (25%)	55 (23%)		32 (24%)	56 (24%)
Hospital Bed Size	Small, n (%)	1 (1%)	1 (1%)		1 (1%)	1 (1%)		6 (5%)	13 (5%)		7 (6%)	13 (5%)
	Medium, n (%)	40 (36%)	18 (20%)	*	31 (28%)	25 (28%)		30 (23%)	41 (17%)		24 (18%)	45 (19%)
	Large, n (%)	70 (63%)	72 (79%)		79 (71%)	64 (71%)		96 (73%)	184 (77%)		100 (76%)	180 (76%)
	N^a^, n (%)	20 (18%)	30 (33%)		28 (25%)	25 (28%)		32 (24%)	29 (12%)		22 (17%)	39 (17%)
Hospital	MW/NC^a^, n (%)	23 (21%)	16 (18%)		21 (19%)	16 (17%)		15 (11%)	47 (20%)	*	22 (17%)	39 (17%)
Region	S^a^, n (%)	58 (52%)	41 (45%)		55 (49%)	42 (47%)		56 (42%)	104 (44%)		54 (41%)	101 (43%)
	W^a^, n (%)	10 (9%)	4 (4%)		8 (7%)	8 (8%)		29 (22%)	58 (24%)		33 (25%)	57 (24%)
Hospital Urban/Teaching (T)	Rural, n (%)	2 (2%)	19 (21%)		12 (11%)	9 (11%)		2 (2%)	5 (2%)		2 (1%)	5 (2%)
	Urban non-T, n (%)	11 (10%)	11 (12%)	*	13 (12%)	10 (11%)		16 (12%)	27 (11%)		15 (11%)	27 (11%)
	Urban T, n (%)	98 (88%)	61 (67%)		87 (77%)	71 (79%)		114 (86%)	206 (87%)		115 (87%)	206 (87%)
